# A Retrospective Study on Genetic Heterogeneity within *Treponema* Strains: Subpopulations Are Genetically Distinct in a Limited Number of Positions

**DOI:** 10.1371/journal.pntd.0004110

**Published:** 2015-10-05

**Authors:** Darina Čejková, Michal Strouhal, Steven J. Norris, George M. Weinstock, David Šmajs

**Affiliations:** 1 Department of Biology, Faculty of Medicine, Masaryk University, Brno, Czech Republic; 2 Department of Immunology, Veterinary Research Institute, Brno, Czech Republic; 3 Pathology & Laboratory Medicine, The University of Texas Health Science Center at Houston, Houston, Texas, United States of America; 4 The Genome Institute, Washington University in St. Louis, St. Louis, Missouri, United States of America; Institut Pasteur, FRANCE

## Abstract

**Background:**

Pathogenic uncultivable treponemes comprise human and animal pathogens including agents of syphilis, yaws, bejel, pinta, and venereal spirochetosis in rabbits and hares. A set of 10 treponemal genome sequences including those of 4 *Treponema pallidum* ssp. *pallidum* (TPA) strains (Nichols, DAL-1, Mexico A, SS14), 4 *T*. *p*. ssp. *pertenue* (TPE) strains (CDC-2, Gauthier, Samoa D, Fribourg-Blanc), 1 *T*. *p*. ssp. *endemicum* (TEN) strain (Bosnia A) and one strain (Cuniculi A) of *Treponema paraluisleporidarum* ecovar Cuniculus (TPLC) were examined with respect to the presence of nucleotide intrastrain heterogeneous sites.

**Methodology/Principal Findings:**

The number of identified intrastrain heterogeneous sites in individual genomes ranged between 0 and 7. Altogether, 23 intrastrain heterogeneous sites (in 17 genes) were found in 5 out of 10 investigated treponemal genomes including TPA strains Nichols (n = 5), DAL-1 (n = 4), and SS14 (n = 7), TPE strain Samoa D (n = 1), and TEN strain Bosnia A (n = 5). Although only one heterogeneous site was identified among 4 tested TPE strains, 16 such sites were identified among 4 TPA strains. Heterogeneous sites were mostly strain-specific and were identified in four *tpr* genes (*tprC*, *GI*, *I*, *K*), in genes involved in bacterial motility and chemotaxis (*fliI*, *cheC-fliY*), in genes involved in cell structure (*murC*), translation (*prfA*), general and DNA metabolism (putative SAM dependent methyltransferase, *topA*), and in seven hypothetical genes.

**Conclusions/Significance:**

Heterogeneous sites likely represent both the selection of adaptive changes during infection of the host as well as an ongoing diversifying evolutionary process.

## Author Summary

The genus *Treponema* comprises several uncultivable human and animal pathogens including *Treponema pallidum* ssp. *pallidum* (TPA), the causative agent of syphilis, *T*. *p*. ssp. *pertenue* (TPE, the causative agent of yaws), and *T*. *p*. ssp. *endemicum* (TEN, the causative agent of bejel). Simian TPE strain Fribourg-Blanc and *T*. *paraluisleporidarum*, the agents of primate infections and venereal spirochetosis of rabbits and hares, respectively, represent animal pathogens. In this study, whole genome sequences of 10 treponemal strains were systematically analyzed for the presence of nucleotide sites where the treponemal strains differed within a single strain. Interestingly, most heterogeneous sites were identified among TPA and TEN strains but not among tested TPE strains. Although heterogeneous sites were found to be mostly strain-specific, several examples revealed the same heterogeneous site was identified in two genomes. These findings indicate that the number of intrastrain heterogeneous sites per genome is limited and that different treponemal strains tend to display variability in the same positions of several genes. The abundance of nonsynonymous mutations, nonconservative amino acid replacements and the fact that most of the heterogeneous sites were located within coding regions suggest that the heterogeneous sites represent beneficial adaptive mutations.

## Introduction

The genus *Treponema* comprises several uncultivable human and animal pathogens including *Treponema pallidum* ssp. *pallidum* (TPA), the causative agent of syphilis, *T*. *p*. ssp. *pertenue* (TPE, the causative agent of yaws), and *T*. *p*. ssp. *endemicum* (TEN, the causative agent of bejel). A treponemal isolate Fribourg-Blanc isolated from a baboon (*Papio cynocephalus*) in West Africa [1],[2] was recently reclassified as a TPE strain [3]. Another animal pathogen closely related to uncultivable human treponemal pathogens is *T*. *paraluisleporidarum* ecovar Cuniculus (TPLC; formerly denoted as *Treponema paraluiscuniculi*) [4–6], the causative agent of venereal spirochetosis in rabbits. In addition, *T*. *paraluisleporidarum* ecovar Lepus [6] causes venereal spirochetosis in hares [7–10]. The human disease pinta is caused by a morphologically identical organism called *T*. *carateum*, but this organism has not been propagated in experimentally infected animals and has not been characterized genetically.

The first complete genome sequence of TPA strain Nichols was determined in 1998 [11]. In the last several years, whole genome sequences of twelve treponemal pathogens (including re-sequenced TPA strains Nichols and SS14) were completed and published [3],[12–20]. In general, genome analyses performed in these studies revealed that genome differences between individual treponemal strains are very subtle, differing in less than 2% of the genome sequence between TPA strains and TPLC [21] and 0.2% between TPA and TPE strains [12]. Genetic diversity among the uncultivable pathogenic treponemes are localized mainly within *tpr* [22–25], *arp* [25–27], TP0470 [25], TP0136 [28],[29], TP0548 [29],[30], *tp92* [31],[32], and *mcp* genes [15]. In addition, relatively high interstrain genetic diversity has been detected in several other genes, e.g. in TP0304 (hypothetical protein), TP0346 (lipoprotein), TP0515 (outer membrane protein), TP0558 (nickel-cobalt transporter) [33] and TP0967 (hypothetical protein) [25].

The presence of different treponemal subpopulations infecting the same host has been suggested by several early findings, e.g. by detection of two subpopulations using velocity sedimentation during the Hypaque separation procedure [34], and by the identification of subpopulation which is resistant to phagocytosis [35]. Genetic diversity within individual treponemal strains, i.e. intrastrain genetic diversity, was first found in *tprJ* and *tprK* genes during infection of human or animal hosts [36–38]. Several other examples of intrastrain heterogeneity were found in the TPA Nichols [21], and in the TPA SS14 genome [14],[16]. In general, intrastrain heterogeneity was found within *tpr* genes, in sequences paralogous to *tpr* genes and in the intergenic regions between *tpr* genes [14],[16],[36–40]. Other genes with identified intrastrain heterogeneity comprised TP0402 (encoding flagellum specific ATP synthase), TP0971 (encoding Tp34 lipoprotein, membrane antigen), TP1029 (encoding hypothetical protein), TP0341 (encoding MurC), and TP0967 (encoding hypothetical protein) loci [14],[16].

The occurrence of genome heterogeneity (including point mutations, insertions or deletions and gain and loss of mobile genetic elements such as plasmids or phages) within strains is common to many pathogenic bacteria [41–44], and has been found to occur during the course of infection [45–51]. In general, heterogeneous sites may contribute to immune evasion [49] and/or represent adaptive changes during infection of disparate host tissues and compartments [52]. The identification of within-host heterogeneity is an important step in studies tracking transmission networks or in studies mapping bacterial populations during colonization, dissemination and immune clearance [53],[54].

In this communication, whole genome sequences of 10 treponemal strains were systematically analyzed for the presence of intrastrain nucleotide heterogeneous sites. Distinct patterns in the frequency and locations of intrastrain heterogeneous sites were identified among the individual genomes examined.

## Materials and Methods

### Strains used in this study

The original sequencing data obtained during next-generation sequencing of pathogenic treponemes (Table 1) were used to analyze intrastrain genetic variability. In total, 10 treponemal strains were examined in this study including 4 TPA strains (Nichols, DAL-1, Mexico A, SS14), 4 TPE strains (CDC-2, Gauthier, Samoa D, Fribourg-Blanc), 1 TEN strain (Bosnia A) and one strain of TPLC (Cuniculi A). For the two remaining whole genome sequences (TPA strains Chicago and Sea84-1), the original sequencing data were not deposited in the SRA database.

**Table 1:** Treponemal genomes used in this study.

Genome	Place and year of isolation	Reference	GenBank Accession number, SRA Accession number (Genome reference)
			Average coverage (Illumina/454), average Illumina read length (bp), estimated Illumina error rate from BWA^a^ (%)
TPA Nichols	Washington, D.C., USA; 1912	[93]	CP004010.2, SRX012305 [16] 31x/30x, 36, 1.65%
TPA DAL-1	Dallas, USA; 1991	[94]	CP003115.1, SRX012302 [18] 38x/33x, 36, 2.07%
TPA SS14	Atlanta, USA; 1977	[95]	CP004011.1, SRX012306 [16] 40x/29x, 36, 1.93%
TPA Mexico A	Mexico City, Mexico; 1953	[96]	CP003064.1, SRX012304 [15] 43x/-, 36, 1.51%
TPE CDC-2	Akorabo, Ghana; 1980	[97]	CP002375.1, SRX012301 [12] 38x/28x, 36, 2.07%
TPE Gauthier	Brazzaville, Congo; 1960	[98]	CP002376.1, SRX104412 [12] 56x/33x, 35, 0.80%
TPE Samoa D	Apia, Samoa; 1953	[96]	CP002374.1, SRX012307 [12] 42x/21x, 36, 2.19%
TPE Fribourg-Blanc	Guinea; 1966	[1],[2]	CP003902.1, SRX104411 [3] 66x/52x, 35, 0.32%
TEN Bosnia A	Bosnia; 1950	[99]	CP007548, SRX144510, SRX144511, SRX144514, SRX144515 [20] 194x/72x, 100, 0.30%
TPLC Cuniculi A	unknown; before 1957	[96]	CP002103.1, SRX012308 [17] 20x/9x, 36, 1.61%

^a^error rate per nucleotide was estimated using the Borrows-Wheeler Aligner (BWA) [55],[56]

To examine intrastrain heterogeneity within a single strain, selected intrastrain heterogeneous sites were tested in the TPA SS14 strain using four different DNA preparations (4933, 4934, 4950 and 4051), originating from two different rabbit passages. The original treponemal SS14 cells were obtained from Dr. D. L. Cox as stock 2735 (dated 09/24/97) and 2736 (dated 06/20/97), which were used to inoculate rabbits and to harvest treponemal cells of stocks 2839 and 2840, respectively. Bacterial stock 2839 of TPA SS14 was used for two independent isolations of genomic DNA using Wizard Genomic DNA Purification Kit (Promega, Madison, WI, USA), resulting in DNA isolates numbered 4933 and 4950. Similarly, bacterial stock 2840 of TPA SS14 was used for two independent isolations of genomic DNA designated as 4934 and 4951. At least one independent rabbit passage between stock 2735 and stock 2736 was performed.

### Ethics statement

No animal was used in the study.

### Identification of intrastrain heterogeneous sites

**Fig 1:**
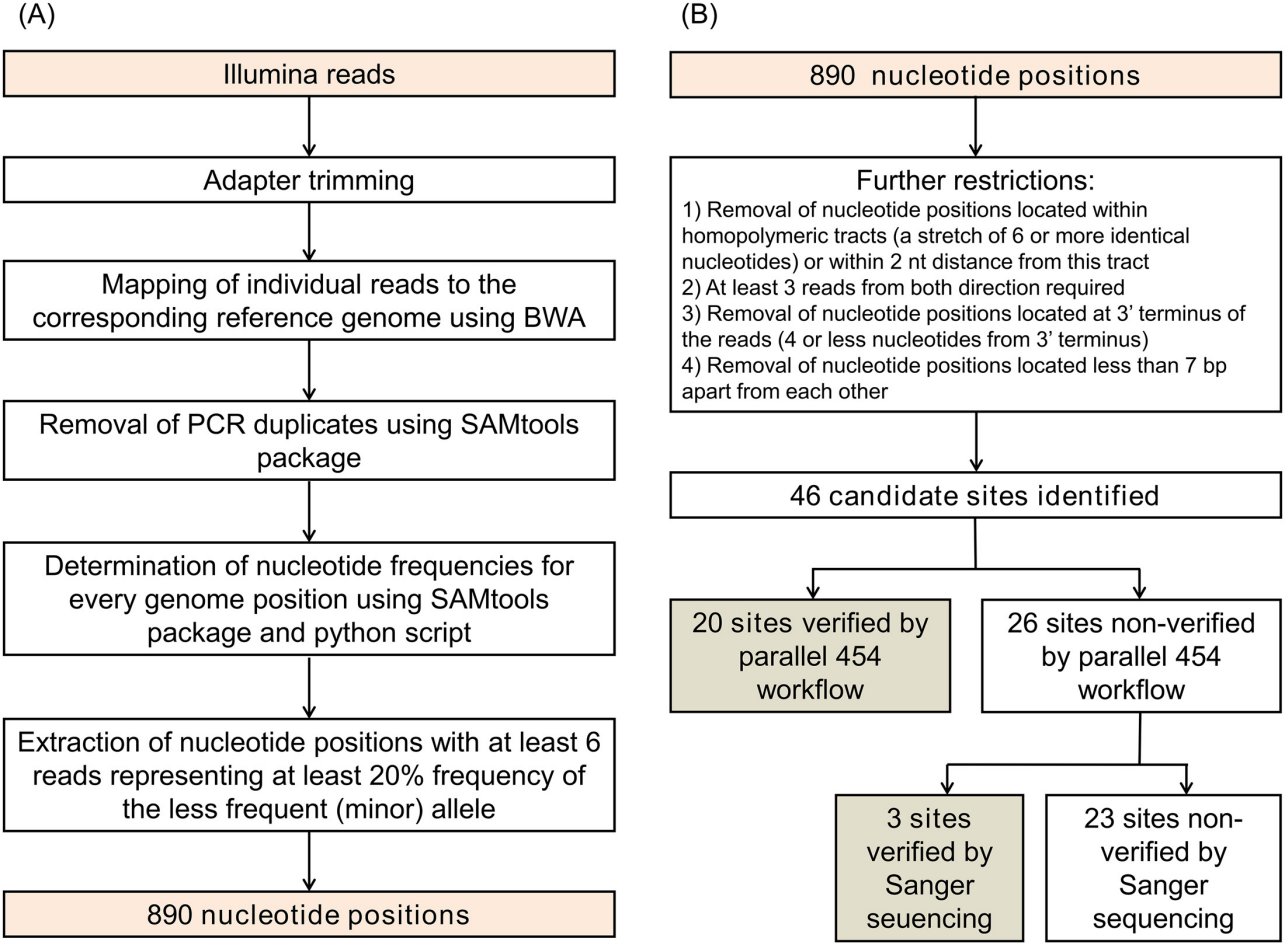
Data analysis workflow. **(A)** An automated identification pipeline and optimization process. **(B)** An application of further restrictions and verification of identified putative candidates.

To ascertain intrastrain heterogeneity within individual treponemal strains, Illumina and 454 reads obtained during whole-genome sequencing procedures were used. Data analysis workflow is depicted in Fig 1. Initially, individual reads were mapped to the corresponding complete genome sequence using the Borrows-Wheeler Aligner (BWA) [55],[56], using default parameters, and requiring at least a 95% read identity relative to the reference genome. Duplicated reads were identified with the rmdup algorithm in the SAMtools package [55] and removed. To determine the frequency of each nucleotide (allele frequency) in every single genome position, the mpileup function in the SAMtools package and a python script were used [57]. Because of higher depth coverage and a lower error indel rate, the Illumina sequencing reads were used for intrastrain allele identifications.

To filter out sequencing errors present in the raw data [58–65], nucleotide positions showing at least six independent (not duplicated) individual reads with a frequency ≥ 20% of the less frequent allele, were further examined. Moreover, several other restrictions were applied during identification of treponemal heterogeneous sites (Fig 1). First, nucleotide positions located within homopolymeric tracts (defined as a stretch of 6 or more identical nucleotides) or within a 2-nt distance of these tracts were omitted from further analysis. Second, at least three independent reads from both directions were required. Third, individual reads supporting a less frequent allele located at the 3’ terminus of the reads (i.e. four or less nucleotides from the 3’ terminus) were omitted. And fourth, heterogeneous positions separated from each other by less than 7 bp were also omitted. The resulting candidate sites for heterogeneous nucleotide positions were subsequently visually inspected using a Integrative Genome Viewer (IGV) [63–66].

Using the above mentioned workflow applied on Illumina reads, putative heterogeneous sites were identified. Identified heterogeneous positions were confirmed using a parallel 454 workflow or by Sanger sequencing (Fig 1 and Table 2 and S2 Table). A detailed description of regions, comprising paralogous sequence regions or/and direct repeats, omitted from Illumina analysis are shown in S1 and S2 Tables. Altogether, 32 genomic regions covering 26,636 bp (2.34% of the entire genome length) were omitted in the TPA Nichols genome (S1 Table). Since paralogous regions in individual genomes are not identical, slightly different regions were omitted from the automated analyses of Illumina sequencing reads in each examined genome (S2 Table). Moreover, the TEN Bosnia A genome was sequenced using pooled segment genome sequencing (PSGS) [12] as separate sequencing runs, therefore the total length of the excluded regions was lower than in other examined genomes (S2 Table).

**Table 2:** Summary of the intrastrain variable sites identified within Illumina sequencing reads in investigated treponemal genomes.

*T*. *p*. strain Average coverage Illumina/454^a^	Genome sequence	Verified by 454 or Sanger sequencing	Major/minor allele	Gene/Genome position	Amino acid change^b^	Protein function/Functional group	Cell localization^c^
**TPA Nichols**	T	454	T/C	TPANIC_0006/7179	*56S; read through stop codon	Hypothetical protein/Unknown	cytoplasm
**31x/30x**	T	454	T/C	TPANIC_0051/59894	S104P	PrfA/Translation	cytoplasm
	A	454	A/C	TPANIC_0222/228259	E46D; conservative	Hypothetical protein/Unknown	unknown
	G	Sanger	G/A	TPANIC_0471/500905	D357N	Hypothetical protein/Unknown	cytoplasmic membrane
	T	454	G/T	upstream of TPANIC_0584/635418	n/a^d^	n/a	n/a
**TPA DAL-1**	C	454	C/T	TPADAL_0065/71972	R70W	SAM dependent methyltransferase/General metabolism	cytoplasm
**38x/33x**	G	Sanger	G/A	TPADAL_0720/789942	A155V; conservative	CheC-FliY/Motility, Chemotaxis	cytoplasm, flagellar
	T	454	T/C	TPADAL_0720/790038	N123S	CheC-FliY/Motility, Chemotaxis	cytoplasm, flagellar
	T	454	T/G	TPADAL_0897/976768	K338Q	TprK/Virulence	periplasm [85]
**TPA SS14**	G	454	G/C	TPASS_20117/135108	N533K	TprC/Virulence	outer membrane [100]
**40x/29x**	A	454	A/G	TPASS_20117/135261	Y483H	TprC/Virulence	outer membrane [100]
	T	454	C/T	TPASS_20341/364888	L64P	MurC/Cell structure	cytoplasm
	A	Sanger	A/C	TPASS_20394/420117	H107P	TopA/DNA metabolism	cytoplasm
	T	454	T/C	TPASS_20402/428628	L134P	FliI/Motility	cytoplasm
	G	454	G/T	TPASS_20402/428930	A235S	FliI/Motility	cytoplasm
	G	454	G/A	TPASS_21029/1125352	D12D; synonymous	Hypothetical protein/Unknown	cytoplasm
**TPE Samoa D**	C	454	C/T	TPESAMD_0134/155544	C284Y	Hypothetical protein/Unknown	unknown
**42x/21x**							
**TEN Bosnia A**	C	454	C/G	TENDBA_0314/331578	E215Q	Hypothetical protein/Unknown	unknown
**194x/72x**	A	454	A/T	TENDBA_0314/331618	H201Q	Hypothetical protein/Unknown	unknown
	A	454	A/G	TENDBA_0316/333355	V240A; conservative	chimeric TprGI^e^/Virulence	unknown
	C	454	C/T	TENDBA_0621/672156	T104T; synonymous	TprI/Virulence	unknown
	S	454	C/G	TENDBA_0897/974407	E347Q	TprK/Virulence	periplasm [69]
	TCCTCCCCC	454	9 bp indel^f^	TENDBA_0967/1049918-1049951	n/a	Hypothetical protein/Unknown	unknown

Illumina-identified intrastrain variable sites were verified using 454 or Sanger sequencing.

^a^no intrastrain heterogeneous site were identified in the TPA Mexico A, TPE CDC-2, TPE Gauthier, TPE Fribourg-Blanc and TPLC Cuniculi A genomes

^b^nonconservative amino acid replacements are not listed

^c^if not indicated, localization was predicted by PSORTb

^d^not applicable

^e^[20],[23]

^f^variable number of direct repeat (TCCTCCCCC)

### DNA amplification and DNA sequencing

**Fig 2:**
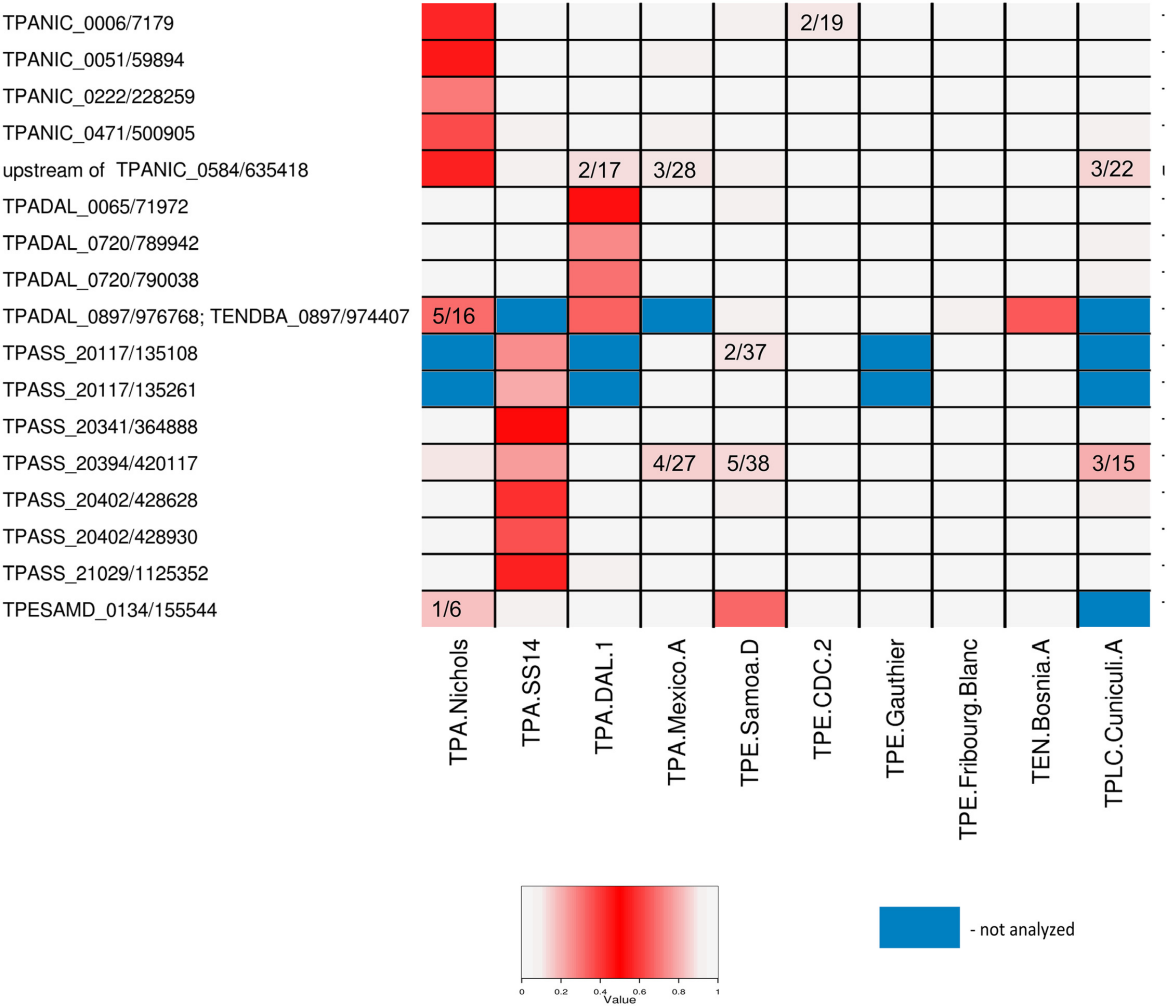
A schematic representation of the identified heterogeneous positions in all investigated genomes. The proportion of alternative alleles is based on nucleotide frequency within individual Illumina reads. While red cells represent identified sites of intrastrain heterogeneity, grey cells represent sites of intrastrain homogeneity. The numbers within cells indicate the number of alternative/standard reads in the sites where the number of alternative reads exceeded 10% but were lower than 20% and therefore remained below the threshold used in this study. Blue cells show nucleotide positions omitted from analysis due to excluded paralogous sequences (S2 Table). For the Bosnia A strain, the intrastrain heterogeneous sites TENDBA_0314/331578, TENDBA_0314/331618, TENDBA_0317/333355 and TENDBA_0621/672156 are not shown because in all other genomes these positions were excluded from analysis due to paralogous sequences. Note that the TPADAL_0897/976678 and TENDBA_0897/974407 positions are the same.

Altogether, 26 putative heterogeneous positions identified in the Illumina workflow, but not confirmed by the 454 sequences (Fig 2, Table 2 and S3 Table) were subjected to DNA amplification and Sanger sequencing. Moreover, six heterogeneous positions identified in the TPA SS14 genome in this study or by Matějková et al. [14] were tested in four different SS14 DNA preparations originating from two different rabbit passages (Table 3). Primers used for DNA amplification and sequencing are specified in S4 and S5 Tables. PCR was performed as follows: initial cycle at 94°C (1 minute), was followed by 30 cycles at 94°C (30 seconds), 55°C (30 seconds), and 72°C (1 minute), and by the final extension step at 72°C (7 minutes). Sequencing of the PCR products was performed using primers used for PCR amplifications with the dye-terminator Sanger sequencing technology. The frequency of alternative alleles in heterogeneous positions was calculated from the ratio of corresponding areas under the chromatogram curves. Sequence analysis of Sanger reads was performed using Lasergene software (DNASTAR, Inc., Madison, WI, USA).

**Table 3:** Selected intrastrain heterogeneous sites identified in TPA SS14, examined in four different DNA preparations.

Bact erial stock no.	DNA preparation no.	G/C^a^ ^,^ ^c^ ^,^ ^d^	A/G^c^ ^,^ ^d^	T/C^c^	T/C^c^ ^,^ ^d^	G/T^c^ ^,^ ^d^	T/C^d^
		TPASS_20117/135108	TPASS_20117/135261	TPASS_20341/364888	TPASS_20402/428628	TPASS_20402/428930	TPASS_20971/1056002
**2839**	4933	G/C (0.0–0.1)	A/G (0.0–0.2)	T/C (0.5–0.6)	T (0.0)	T (1.0)	T/C (0.5–0.6)
	4950^b^	G (0.0)	A (0.0)	T/C (0.5–0.6)	T (0.0)	T (1.0)	T/C (0.7)
**2840**	4934	G/C (0.3–0.4)	A/G (0.4–0.6)	T/C (0.7)	T/C (0.2–0.3)	G/T (0.4–0.7)	T/C (0.3)
	4951^b^	G/C (0.3–0.4)	A/G (0.4–0.5)	T/C (0.5)	T/C (0.3–0.4)	G/T (0.3–0.6)	T/C (0.1)

DNA preparations originated from two different rabbit passages. Relative proportions of alleles not stated in the reference genome are shown in parentheses as derived from repeated Sanger sequencing.

^a^the first nucleotide corresponds to the sequence published in the SS14 genome sequence CP004011.1 [16]

^b^DNA preparations 4950 and 4951 were used for whole genome sequencing of the TPA SS14 strain by Matějková et al. [14]; preparation 4951 was used for re-sequencing of this strain [16]

^c^heterogeneous positions identified in this study (Table 2)

^d^heterogeneous positions identified by Matějková et al. [14]

### Conserved protein domain database search

The NCBI Conserved Domain Database [67] and InterProScan [68] were used to predict protein domains. Putative protein localization within a cell was determined using the PSORTb program [69].

## Results

### Identification of intrastrain heterogeneous sites

A set of 10 treponemal whole genome sequences including those of 4 TPA strains (Nichols, DAL-1, Mexico A, SS14), 4 TPE strains (CDC-2, Gauthier, Samoa D, Fribourg-Blanc), 1 TEN strain (Bosnia A) and one strain of TPLC (Cuniculi A) were examined with respect to the presence of intrastrain heterogeneous sites. All but one (TPA Mexico A) genomes were sequenced using both Illumina and 454 sequencing methods. Characteristics of the sequence data obtained with each strain, including the average coverage attained during Illumina and 454 sequencing, are shown in Table 1. Altogether, 890 potentially heterogeneous positions among investigated genomes were identified using an automated pipeline (Fig 1). Several criteria (see Materials and methods) were used to filter out sequencing errors from genetic heterogeneity naturally occurring in treponemal strains (i.e. representing intrastrain heterogeneous sites), which reduced the 890 nucleotide positions to 46 candidates (Fig 1). Regions containing paralogous sequences and tandem repeats (summarized in S1 and S2 Tables) were omitted from the automated analyses of intrastrain heterogeneity due to the risk of ambiguously mapped reads. Using these criteria, 32 genomic regions covering 26,636 bp (2.34% of the entire genome length) were excluded from the analysis of Illumina sequencing reads in the TPA Nichols genome (S1 Table). Except for the TEN strain Bosnia A, similar regions were also excluded in whole genome sequences in other tested genomes (S2 Table) (see Materials and Methods).

An instance of intrastrain heterogeneity was considered to be present if 1) two different nucleotides (or an indel) were detected at a given genome coordinate, and 2) this heterogeneity was present in at least two sequencing analyses using different sequencing chemistry. The automated analysis of Illumina reads revealed 46 candidates (Fig 1), of which 20 heterogeneous sites were directly verified by automated analysis of 454 reads. The remaining 26 candidate sites, solely found in Illumina reads, were sequenced using Sanger technology, and in three of them, heterogeneous sites were identified (Tables 2 and S3).

### Intrastrain heterogeneous sites are mainly present in TPA and TEN but not in TPE strains

The 23 intrastrain heterogeneous sites, identified using the automated analysis of Illumina sequencing reads and either 454 or Sanger sequencing reads, were found in 5 out of 10 investigated treponemal genomes (Table 2), including TPA strains Nichols, DAL-1, and SS14, TPE strain Samoa D and TEN strain Bosnia A. No intrastrain heterogeneous sites were identified in TPA Mexico A, TPE CDC-2, Gauthier, Fribourg-Blanc and TPLC Cuniculi A genomes. Up to 7 intrastrain heterogeneous sites were identified in individual genomes. Whereas only one heterogeneous site was identified in the 4 examined TPE strains, 16 heterogeneous sites were detected among the 4 TPA strains analyzed. The TEN strain Bosnia A contained 5 single nucleotide heterogeneous sites, however, four of these heterogeneous sites (TENDBA_0314/331578, TENDBA_0314/331618, TENDBA_0317/333355 and TENDBA_0621/672156) were located within paralogous regions that had been excluded from analysis in all other genomes (S2 Table). In contrast to other genomes, the TEN Bosnia A genome was sequenced using the pooled segment genome sequencing method (PSGS) [20] as four distinct samples, whereas other treponemal genomes were not subdivided prior to Illumina sequencing. Therefore, orthologous genes to TENDBA_0314, TENDBA_0317 and TENDBA_0621 genes were not completely analyzed in other genomes. In contrast, the same heterogeneous site found in the *tprK* gene of TEN Bosnia A (TENDBA_0897/974407) was also identified in the TPA DAL-1 strain (TPADAL_0897/976768). Interestingly, this genome position is included in *tprK* variable regions of the TPA SS14 and Mexico A genomes, however, it was included in non-variable regions in all other genomes [37]. Therefore, in TPA SS14 and Mexico A genomes, these *tprK* hypervariable regions were excluded from analyses (Fig 2). In four cases, comprising genes TPASS_20117 (*tprC*), TENDBA_0314 (hypothetical gene), TPASS_20402 (*fliI*) and TPADAL_0720 (*fliY)*, two heterogeneous sites were found in each gene (Fig 2 and Table 2).

### Characteristics of identified intrastrain heterogeneous sites

All but one heterogeneous sites represented alternative nucleotides resulting from substitutions, while one indel-variable site was found (Table 2). Out of 23 identified heterogeneous sites, one was localized in an intergenic region and all others (n = 22) were within the predicted coding regions comprising 17 genes. The heterogeneous genes encode Tpr proteins (TprC, TprI, TprK and a chimeric TprGI), proteins involved in bacterial motility and chemotaxis (FliI and CheC-FliY), translation proteins (PrfA), peptidoglycan synthesis (MurC), general metabolism (putative SAM dependent methyltransferase), DNA metabolism (TopA), and hypothetical proteins of unknown function (TPANIC_0006, TPANIC_0222, TPANIC_0471; TPASS_21029; TPESAMD_0134; TENDBA_0314, TENDBA_0967).

One alternative allele resulted in replacement of a stop codon and resulted in protein elongation, while the others resulted in synonymous (n = 2) or nonsynonymous mutations (n = 18). Of the nonsynonymous mutations, 3 resulted in conservative and 15 in nonconservative amino acid replacements (Table 2). Transitions (n = 13) were found more frequently than transversions (n = 9). Most frequent were C→T and G→A (n = 9) transitions while T→C and A→G transitions were less frequent (n = 4). C→A and T→A transversions were not found.

### Identification of the intrastrain heterogeneous sites in different passages of TPA SS14

To test whether intrastrain heterogeneous sites were present stably within different rabbit passages, a set of intrastrain heterogeneous sites identified in the TPA SS14 were examined in four different DNA preparations originating from two different rabbit passages (see Materials and methods, Table 3). While DNA samples 4933 and 4950 were isolated from the same batch of treponemal cells (batch 2839), DNA samples 4934 and 4951 were prepared from bacterial stock 2840. Only minimal differences in the presence and frequency of alternative alleles were found between 4933 and 4950 (and also between 4934 and 4951), whereas clear differences between DNA preparations obtained from bacterial stocks 2839 and 2840 were found (Table 3).

## Discussion

In this study, correct identification of intrastrain variable sites was considered of critical importance. To filter out sequencing errors, several restrictions in detecting algorithms were applied. Paralogous genome regions were omitted from analyses due to the risk of incorrect mapping of individual reads belonging to different genome regions. Duplicated reads, i.e. reads that showed identical start and end points were automatically identified and removed from further analyses in order to analyze only uniquely generated sequencing reads and to remove potential bias during DNA amplification. Since most of the Illumina errors are nucleotide substitutions located at the 3’ DNA end [58],[70], sequence differences close to the 3’ DNA end (at positions that were 4 or less nucleotides from end) of individual reads were filtered out. An increased error rate, within and in close proximity to homoplymeric regions, was also reported in the original Solexa chemistry [71]. Therefore, we also filtered out differences in homopolymeric tracts and in close vicinity (defined as 2-nt distance) to homopolymeric tracts although we are aware that the variations in length of homopolymeric tracts, especially those composed of guanosine tandem repeats, are of biological importance. These tandem repeats are known to regulate transcription (if located in promoter regions) and have been identified in the *T*. *pallidum* genomes [72],[73]. To further increase validity of the results, only alternative reads reaching at least a 20% frequency were analyzed. In summary, these relatively stringent measures certainly led to a number of missed heterogeneous sites both in the analyzed and in the non-analyzed genome regions. In addition to missed single nucleotide heterogeneous sites, larger sequences showing genetic heterogeneity were likely also missed due to the relatively short length of Illumina reads and due to applied restrictions in the detection algorithm. An example of such sites could be the 1.3 kb-long *tprK*-like sequence between TP0126 and TP0127 or the 64 bp-long indel between TP0135 and TP0136, previously identified in the TPA Nichols genome [25],[39]. Another example comes from this work where one region of intrastrain heterogeneity comprising a 9 nt-long insertion sequence in TENDBA_0967 was found in the Bosnia A strain during manual inspection of individual reads. The insertion represents an additional tandem repetition within a larger region between coordinates 1044918 and 1044951. Despite the possibility of missed sites of intrastrain heterogeneity, the automated analysis pipeline used in this study revealed 46 putative heterogeneous sites and 23 of them (50.0%) were verified using an independent sequencing method with different sequencing chemistry. The remaining, non-verified 23 positions likely represent falsely identified sites, likely as a consequence of accumulated error-containing Illumina reads. The majority of heterogeneous sites identified in this study represented transitions and not transversions, which, in general, are common Illumina sequencing errors; A→C was most common, followed by G→T transversions [59],[70]. The number of heterogeneous sites in a particular genome did not correlate with average sequencing coverage nor with estimated percent Illumina error rate per nucleotide (Table 1).

Although heterogeneous sites were found to be mostly strain-specific, several examples revealed the same heterogeneous site was identified in two genomes. The same heterogeneous site was found in the *tprK* gene of the DAL-1 and Bosnia A genomes. Interestingly, the same position was also found to be heterogeneous in the Nichols genome, although the number of Illumina reads supporting the less frequent nucleotide remained below threshold (SRX012305, Fig 2). A similar situation was also found in two other sites, one in SS14 and Cuniculi A genomes and the other one in Samoa D and Nichols genomes (Fig 2). These findings indicate that the number of intrastrain heterogeneous sites per genome is limited and that different treponemal strains tend to display variability in the same positions of several genes. The abundance of nonsynonymous mutations, nonconservative amino acid replacements and the fact that most of the heterogeneous sites were located within coding regions suggest that the heterogeneous sites represent beneficial adaptive mutations [74].

In this study, 23 intrastrain heterogeneous sites in 17 genes were identified in 5 out of 10 investigated treponemal genomes, predominantly in TPA strains. The reason why most of the heterogeneous sites were identified in the TPA, but not in TPE strains, is not clear, however, it might reflect different tissue tropism of TPA and TPE strains, different growth rate in experimental rabbits, differences in pathogenesis or other reasons. Regardless, this finding indicates distinct genetic characteristics of TPA and TPE strains. Although the TEN strain Bosnia A resembled TPA strains in this respect, most of the heterogeneous positions were identified in paralogous regions which were excluded from the automated analysis of other genomes (Fig 2). The single heterogeneous site identified in nonparalogous regions in the Bosnia A genome thus resembles TPE strains. In fact, the Bosnia A genome is more related to TPE strains than to TPA strains, although several sequences similar to TPA sequences were identified in the Bosnia A genome [20]. In contrast to other TPA strains, analysis of the TPA Mexico A strain did not reveal any heterogeneous sites (Fig 1 and Table 2). Unlike other TPA strains, the Mexico A genome has been shown to contain two TPE-like sequences [15]. However, it remains unclear whether these two observations are related.

A comparison of our results with a previously published paper describing heterogeneous sites in the TPA SS14 strain [14] is shown in the Table 4. In the analyzed portion of the SS14 genome, Matějková et al. found 18 heterogeneous sites. Out of these 18 sites, we automatically detected 5 sites. In other 4 sites, the frequency of the alternative allele was below threshold and/or did not meet restriction criteria, nonetheless manual inspection revealed the presence of the alternative allele. In additional two cases, the heterogeneity was identified in 454 reads (SRX000109), but not by Illumina reads. Comparison of our results with those published by Matějková et al. [14] identified a substantial overlap, however, 7 sites (38.9%) detected by Matějková et al. were not found in our study. Interestingly, all non-detected heterogeneous sites were located in *tpr* genes (including *tprC*,*I*,*J*) or in the intergenic regions between them. At least two independent explanations can be proposed; one explanation involves the fact that the BWA (Borrows-Wheeler Aligner) mapping algorithm used in this study was not able to detect closely spaced heterogeneous sites representing a specific haplotype in relatively short Illumina or 454 reads, due to alignment restrictions. To align an individual read to the reference sequence, a 95% identity with the reference genome sequence was required in our study. However, no such reads were found in the raw data set (SRX012306, SRX000109). The other explanation involves falsely identified heterogeneous sites as a result of PCR-based errors introduced during amplification of diluted target DNA and subsequent cloning of PCR products, as was done in the work of Matějková et al. [14]. The latter explanation is also supported by the fact that the undetected heterogeneous sites were often supported by low numbers of alternative clones (Table 4). Deeper sequencing of identified heterogeneous genome sites will be needed to answer these questions.

**Table 4:** Comparison of heterogeneous positions identified in TPA SS14 strain by Matějková et al. [14] and by the automated pipeline used in this study.

Gene	Genome position in the SS14 genome CP000805.1 (CP004011.1)^a^	Heterogeneity identified by Matějková et al. [14]^b^	Nucleotide frequency identified in this study^b^	Heterogeneity detected in Illumina reads
TPASS_20117	135098 (135108)	G or C (5/6)	G or C (32/12)	yes
	135107 (135117)	T or C (3/4)	T or C (50/1)	Yes^c^
	135235 (135245)	G or A (2/10)	A (46)	no
	135239 (135249)	C or T (2/10)	T (49)	no
	135251 (135261)	A or G (6/6)	A or G (41/11)	yes
TPASS_20402	427435 (428628)	C or T (NA)	C or T (15/21)	yes
	427737 (428930)	G or T (NA)	G or T (25/14)	yes
TPASS_20620	671746 (673228)	T or C (9/3)	T (23)	no
	671751 (673233)	T or G (19/10)	T (22)	no (but detected by 454)^d^
	671753 (673235)	T or C (19/10)	T (22)	no (but detected by 454)^d^
	671763 (673245)	C or T (8/4)	C or T (24/5)	yes^c^ (also detected by 454)^d^
	672286 (673768)	G or A (4/12)	A (29)	no
Upstream of TPASS_20620	672916–7 (674399–674400)	(-) or C (6/6)	(-) or C (7/5)	yes^c^
	672944 (674427)	A or G (14/6)	A (14)	no
TPASS_20621	673425 (674908)	C or T (2/8)	T (44)	no
	673428 (674911)	A or G (2/8)	G (44)	no
TPASS_20971^e^	1054447 (1056002)	T or C (NA)	T or C (35/3)	yes^c^
TPASS_21029	1123796 (1125352)	G or A (5/6)	G or A (24/18)	yes

^a^additional intrastrain heterogeneous genome positions identified by Matějková et al. [14] including 135141, 135144, 135149, 135220, 135227, 671982, 672004, 672016, 672025, 672026, 672027, 672028, 672036, 672039, 672040, 672041, 672042, 672043, 672044, 672154, 673088, 673119, 673511, 673545, 673550, and 673554 (according to the CP000805.1) were located in paralogous regions and therefore were excluded from the automated pipeline (S2 Table)

^b^numbers in parentheses show numbers of sequenced clones [14] or nucleotide frequency within individual Illumina sequence reads (this study); NA—not available

^c^not present in Table 2; heterogeneous positions were detected in raw Illumina sequencing reads but were excluded due to study criteria

^d^ these heterogeneous sites were not found among Illumina reads, but were identified among 454 reads (SRX000109)

^e^see also Table 3; independent DNA preparations showed clear differences in proportions of alternative alleles, ranging from 0.1 to 0.7

In bacterial genomes, most mutations represent C→T transitions arising via deamination of cytosine [75], T→C transitions via oxidation of thymine and/or inefficient DNA repair [76], A→G transitions via deamination of adenine [76], and G→T transversions via oxidization of guanine [76]. In fact, these 4 (out of 12 possible) mutations were observed in 11 out of 22 single nucleotide substitutions (50%) indicating that most common types of substitutions overlap with the most frequently seen bacterial mutations. In contrast, sample oxidation frequently results in C→A and G→T changes [77], while Illumina errors are predominantly A→C transversions [59],[70]. Only three such substitutions (out of 22; 13.6%) were, in fact, found in this study indicating that these substitutions are not overrepresented. Interestingly, the candidate sites identified using the Illumina pipeline, but not verified by other sequencing techniques (S3 Table), frequently (in 73.9%) included these types of mutations, which points to Illumina as a source of errors and false-positive results.

TPA SS14 bacterial stocks 2839 and 2840 differed in at least 12–14 treponemal generations of separated cultivation corresponding to two rabbit subcultivations each, of approximately 100-fold increase, in the number of treponemes per subcultivation. Heterogeneous sites were clearly different in DNA preparations obtained from different bacterial stocks, indicating the dynamic nature of this heterogeneity. This observation could also explain the strain-specificity of intrastrain heterogeneous sites identified in this study. The role of rabbit passages in the occurrence of heterogeneous sites remains unknown, however, genetic heterogeneity has also been identified in treponemes isolated directly from human host (Natasha Arora, personal communication). The occurrence of intrastrain heterogeneity in TPA from human samples suggests its potential significance for molecular typing of syphilis treponemes by both sequencing approach [78],[79] and RFLP analysis of amplified genes [80],[81].

Out of 22 heterogeneous sites showing alternative nucleotides, 16 heterogeneous sites were found in conserved genome positions (where all investigated genomes had identical sequences), while 6 were found in genome positions in which the analyzed genomes differed in sequence. In 5 out of 6 sites, alternative nucleotides of heterogeneous positions matched nucleotide sequences present in analyzed genomes. Considering the highest divergence observed in treponemal genomes, which represents 0.84% sequence diversity between the conserved regions of the TPA and TPLC genomes [17], the theoretical probability that a heterogeneous site would be located at a nonconserved genome position is 8.4 x 10^−3^. In our study, heterogeneous sites were found more frequently (in 6 out of 22) in nonconserved genome positions (2.7 x 10^−1^; p < 0.001), suggesting the role of heterogeneous sites in the process of treponemal genome diversification.

This study identified heterogeneous sites in four *tpr* genes, in genes involved in bacterial motility and chemotaxis (2), in cell structure (1), translation (1), general and DNA metabolism (2), and in seven hypothetical genes. The average expression rate of these 17 genes (1.33) during experimental rabbit infection was greater than the whole genome average (1.0) [82] indicating that these genes are expressed during host infection. Interestingly, heterogeneous sites were identified in *tprC*, *tprI*, *tprK* and chimeric *tprGI* genes. Several studies have shown that Tpr antigens are expressed during infection and are able to elicit antibody and cellular immune responses in the infected host [23],[83],[84]. Moreover, several Tpr proteins have been predicted to be outer membrane proteins [23],[85]. In addition, the *tprK* gene undergoes antigenic changes in seven variable regions and TprK variants are selected by the immune response [86],[87]. It has also been shown that *tprK* variants accumulate during infection of the host [88],[89] and that individual TprK variants helped to disseminate *T*. *pallidum* infections [87]. As demonstrated by LaFond et al. [90], variable regions elicited a variant-specific antibody response indicating that minor sequence changes may affect antibody binding. In this context, nonconservative changes could result in strain-specific surface-exposed epitopes that are crucial for immune evasion as previously predicted for discrete variable regions within TprC and TprD [23]. In *E*. *coli*, the *topA* (corresponding to TPASS_20394) mutation has been shown to affect fitness relative to isogenic constructs [91]. Moreover, *topA* and genes involved in cell wall biosynthesis and translation have been shown to repeatedly mutate in independent lines of *E*. *coli* during long-term cultivation experiment [74]. Heterogeneous sites in pathogenic treponemal strains may therefore represent adaptive changes that take place during infection of various host tissues and compartments as described in other bacteria [52]. At the same time, these sites may represent snapshots of an ongoing evolutionary trajectory. Advances in deep sequencing techniques and prospective whole genome sequencing or metagenomic studies will help, in the future, to identify a larger and perhaps more complete set of treponemal intrastrain heterogeneous sites [53],[54],[92].

## Supporting Information

S1 Table Chromosomal paralogous regions not included in the automated analysis of Illumina sequencing reads of the TPA Nichols genome. (XLS)

S2 Table Chromosomal paralogous regions not included in the automated analyses of Illumina sequencing reads of all investigated genomes. (XLS)

S3 Table A set of 23 putative heterogeneous positions identified solely by the Illumina workflow, but not verified by other sequencing methods. (XLS)

S4 Table Primers used for DNA amplification and Sanger sequencing of 26 heterogeneous candidate positions (not-verified by 454 workflow). (XLS)

S5 Table List of primers used for DNA amplification and Sanger sequencing of selected intrastrain heterogeneous sites in four different TPA SS14 DNA preparations. (XLS)
